# A Single Vector Platform for High-Level Gene Transduction of Central Neurons: Adeno-Associated Virus Vector Equipped with the Tet-Off System

**DOI:** 10.1371/journal.pone.0169611

**Published:** 2017-01-06

**Authors:** Jaerin Sohn, Megumu Takahashi, Shinichiro Okamoto, Yoko Ishida, Takahiro Furuta, Hiroyuki Hioki

**Affiliations:** 1 Department of Morphological Brain Science, Graduate School of Medicine, Kyoto University, Yoshida-Konoe-cho, Sakyo-ku, Kyoto, Japan; 2 Division of Cerebral Circuitry, National Institute for Physiological Sciences, Higashiyama, Myodaiji-cho, Okazaki, Japan; University of Tuebingen, GERMANY

## Abstract

Visualization of neurons is indispensable for the investigation of neuronal circuits in the central nervous system. Virus vectors have been widely used for labeling particular subsets of neurons, and the adeno-associated virus (AAV) vector has gained popularity as a tool for gene transfer. Here, we developed a single AAV vector Tet-Off platform, AAV-SynTetOff, to improve the gene-transduction efficiency, specifically in neurons. The platform is composed of regulator and response elements in a single AAV genome. After infection of Neuro-2a cells with the AAV-SynTetOff vector, the transduction efficiency of green fluorescent protein (GFP) was increased by approximately 2- and 15-fold relative to the conventional AAV vector with the human cytomegalovirus (CMV) or human synapsin I (SYN) promoter, respectively. We then injected the AAV vectors into the mouse neostriatum. GFP expression in the neostriatal neurons infected with the AAV-SynTetOff vector was approximately 40-times higher than that with the CMV or SYN promoter. By adding a membrane-targeting signal to GFP, the axon fibers of neostriatal neurons were clearly visualized. In contrast, by attaching somatodendritic membrane-targeting signals to GFP, axon fiber labeling was mostly suppressed. Furthermore, we prepared the AAV-SynTetOff vector, which simultaneously expressed somatodendritic membrane-targeted GFP and membrane-targeted red fluorescent protein (RFP). After injection of the vector into the neostriatum, the cell bodies and dendrites of neostriatal neurons were labeled with both GFP and RFP, whereas the axons in the projection sites were labeled only with RFP. Finally, we applied this vector to vasoactive intestinal polypeptide-positive (VIP+) neocortical neurons, one of the subclasses of inhibitory neurons in the neocortex, in layer 2/3 of the mouse primary somatosensory cortex. The results revealed the differential distribution of the somatodendritic and axonal structures at the population level. The AAV-SynTetOff vector developed in the present study exhibits strong fluorescence labeling and has promising applications in neuronal imaging.

## Introduction

Elucidating the principal design of neuronal circuits is fundamental for understanding how the brain works and implements higher-order functions [1–5]. Visualization of neurons is the first key step in dissecting the neuronal circuits, and the Golgi silver-staining technique has made a great contribution in this field [6, 7]. In addition to Golgi staining, anterograde and retrograde neuronal tracers, such as *Phaseolus vulgaris* leucoagglutinin (PHA-L) [8], biocytin [9], horseradish peroxidase (HRP) [10], and cholera toxin B subunit (CTb) [11], have prompted the study of neuronal connections in the central nervous system [12–14]. However, these conventional methods have drawbacks such as allowing only random but not specific visualization and incomplete labeling of the targeted neurons owing to technical limitations.

Genetic engineering techniques have been applied to neuroscience, and have afforded specific and sufficient labeling of neuronal subpopulations [15, 16]. Transgenic animal lines that express fluorescent proteins have been utilized widely for labeling particular subsets of neurons [17, 18]. The *in utero* electroporation technique enables one to perform cell birthday-specific labeling by introducing plasmids that contain sequences of reporter proteins into target areas of the brain [19–21]. Virus vectors have aroused increasing interest in the field of basic and clinical neurosciences, since the vectors can be directly and locally delivered to the brain region of interest [22, 23]. In addition, gene expression can be limited to particular types of neurons in combination with Cre-driver mouse lines [24, 25]. These genetic tools can be used to determine the architecture of neuronal networks.

Adeno-associated virus (AAV) vectors are now widely applied for gene delivery to neuronal cells. They can be easily purified and highly concentrated, and gene-expression by AAV vectors is efficient and persistent without pathology [26–28]. However, when using ubiquitous promoters, including the human cytomegalovirus (CMV) promoter, gene expression was observed not only in neuronal cells but also in glial cells [29, 30]. Although neuron-specific promoters, such as the human synapsin I (SYN) promoter, achieve specific expression in neuronal cells, the expression level was 10-times less than that achieved with the CMV promoter *in vitro* with self-complementary AAV9 vectors [31]. Thus, it is necessary to develop an efficient neuron-specific gene-expression system with AAV vectors.

In our previous studies, we applied the “Tet-Off system” to lentivirus vectors, and succeeded in achieving strong gene transduction, specifically in neuronal cells, *in vivo* [32, 33]. The expression system is composed of two kinds of units: 1) the regulator unit expresses an improved version of tetracycline-controlled transactivator (tTAad), specifically in neurons, *via* the SYN promoter, and 2) the response unit strongly expresses the gene of interest under the tetracycline-responsive element (TRE) promoter, to which tTAad binds. In the present study, we incorporated these two units into the single genome of AAV serotype 2 (AAV2), produced virus particles pseudotyped with the capsid from serotype 1, and demonstrated high-level expression of the reporter protein *in vitro* and *in vivo*.

We further prepared AAV2/1 vectors that express green fluorescent protein (GFP) or monomeric red fluorescent protein (mRFP1) [34] with a membrane-targeting signal, such as palmitoylation and/or myristoylation signals [35–39], and clearly labeled the axon fibers of neostriatal neurons. By fusing a dendritic membrane-targeting signal to GFP [39, 40], we visualized the cell bodies and dendrites of the infected cells but not the axonal structures. Finally, we generated the AAV2/1 vector that expresses both somatodendritic membrane-targeted GFP and membrane-targeted mRFP1 in the presence of Cre recombinase, and quantitatively analyzed the dendritic and axonal distributions of vasoactive intestinal polypeptide-positive (VIP+) inhibitory neurons into layer (L) 2/3 of the mouse primary somatosensory cortex barrel field (S1BF).

## Materials and Methods

### Animals

All animal experiments were conducted in accordance with the National Institutes of Health Guide for the Care and Use of Laboratory Animals, and the experiments were approved by the Committee for Animal Care and Use (MedKyo 15012 and MedKyo 16573) and the Committee for Recombinant DNA Study (120093 and 141008) of Kyoto University. Adult male C57BL/6J mice (Japan SLC, Hamamatsu, Japan) and male Vip^tm1(cre)Zjh^/J (VIP-Cre) mice (The Jackson Laboratory, Bar Harbor, ME; stock number 010908; 2–3 months old) [24] were used in the present study. All efforts were made to minimize animal suffering and the number of animals used.

### Plasmid construction for AAV vectors

We amplified the human CMV promoter (nucleotides 1–589 in GenBank U57609.1; primer set P1/P2) or the human SYN promoter (nucleotides 1889–2289 in GenBank accession no. M55301.1; primer set P3/P4 in S1 Table) [41], GFP (primer set P5/P6), and a polyadenylation signal derived from the bovine growth hormone gene (BGHpA; nucleotides 1771–1995 in GenBank AH009106.2; primer set P7/P8) by polymerase chain reaction (PCR). We then inserted the PCR products into the *Hinc*II, *EcoR*V, and *EcoR*I*/BamH*I sites of pBluescript II SK (+) (pBSIISK; Stratagene, La Jolla, CA), respectively, resulting in pBSIISK-CMV-GFP-BGHpA or pBSIISK-SYN-GFP-BGHpA. The sequence of the SYN promoter mostly corresponds to the commonly used one with other virus vectors such as the adenovirus vector [42, 43]. To generate a Gateway entry vector, the *Xho*I-to-*BamH*I fragment from the two plasmids were complementarily inserted into the *BamH*I*/Xho*I sites of pENTR^™^1A (Life Technologies, Carlsbad, CA), resulting in pENTR1A-CMV-GFP-BGHpA(r) or pBSIISK-SYN-GFP-BGHpA(r). We newly prepared destination vectors, pAAV2-DEST(f) and pAAV2-DEST(r), by amplifying the sequence of R1-ccdB-R2 (primer set P9/P10 and P11/P12) from pLenti6 (Life Technologies) and by inserting the fragment into the *Mul*I*/PmaC*I sites of pAAV-MCS (Stratagene). Then, the inserts from the two entry vectors were transferred to the destination vector pAAV2-DEST(r) by homologous recombination with LR clonase II (Life Technologies), resulting in pAAV2-CMV-GFP-BGHpA and pAAV2-SYN-GFP-BGHpA.

In the previous study, we inserted the SYN promoter, tTAad (Clontech, Palo Alto, CA) and BGHpA (amplified by PCR) into pBSIISK, resulting in pBSIISK-SYN-tTAad-BGHpA [32]. GFP and BGHpA (amplified by PCR) were subcloned into pTRE-Tight (Clontech), and the resulting constructs were named as pTRE-GFP-BGHpA [32]. In the present study, we prepared new vectors by modifying these two plasmids as follows. A polyadenylation signal of Simian virus 40 late (SV40LpA) was amplified by PCR (primer set P13/P14), and was replaced with BGHpA in pBSIISK-SYN-tTAad-BGHpA through the *Bgl*II/*Not*I sites, resulting in pBSIISK-SYN-tTAad-SV40LpA. The insulator sequence [44] was inserted into the *BamH*I/*Xho*I sites of pENTR^™^1A (primer set P15/P16), and the resulting construct was named as pENTR1A-insulator. The fragment SYN-tTAad-SV40LpA was amplified by PCR (primer set P17/P18), and then complementarily inserted into pENTR1A-insulator between the *BamH*I/*Sal*I sites, resulting in pENTR1A-SV40LpA-tTAad-SYN-insulator. The fragment TRE-GFP-BGHpA [32] was inserted into the *Xho*I/*Not*I sites of pENTR1A-SV40LpA-tTAad-SYN-insulator, resulting in pENTR1A-SV40LpA-tTAad-SYN-insulator-TRE-GFP-BGHpA.

Through the *BamH*I/*Mlu*I sites in this entry vector, the GFP sequence was replaced with the following sequences of reporter proteins: 1) GFP with a palmitoylation signal derived from GAP-43 N-terminus (palGFP) (primer set P19/20) [32, 35–39]; 2) GFP with a myristoylation/palmitoylation signal derived from Fyn N-terminus (myrGFP) (primer set P21/20) [39, 40]; or 3) myrGFP with a somatodendritic-targeting signal, the C-terminal cytoplasmic domain of low-density lipoprotein receptor (LDLRct), which was originally named as myrGFP-LDLRct in the previous reports (primer set P21/P22) [39, 40], but referred to as FGL in the present study. By using an LR recombination reaction with these entry vectors and a destination vector, pAAV2-DEST(f), we produced pAAV2-SynTetOff-GFP, pAAV2-SynTetOff-palGFP, pAAV2-SynTetOff-myrGFP, and pAAV2-SynTetOff-FGL.

We also prepared a new reporter protein that contained both FGL and mRFP1 [45] tagged with a palmitoylation signal derived from GAP-43 N-terminus (palmRFP1) by overlap PCR as follows: 1) the FGL sequence was amplified with the addition of a furin cleavage site and a 2A self-processing sequence (F2A) [46] to the 3′-terminus (primer set P21/P23); 2) the sequence of palmRFP1 was amplified with the addition of the F2A sequence to the 5′-terminus (primer set P24/P25); and 3) the FGL-2A-palmRFP1 sequence was finally amplified (primer set P21/P25). The PCR products were inserted into pENTR1A-SV40LpA-tTAad-SYN-insulator-TRE-GFP-BGHpA through the *BamH*I/*Mlu*I sites. After an LR recombination reaction with the entry vector and a destination vector, pAAV2-DEST(f), we obtained pAAV2-SynTetOff-FGL-2A-palmRFP1.

For specific gene expression under Cre exposure, we used the flip-excision (FLEX) switch [47]. The switch sequence, composed of two pairs of *loxP* and *lox2272* sites in opposite orientations, was synthesized *de novo* (S1 Table; GenScript, Piscataway, NJ) and inserted into pBSIISK through the *Kpn*I/*Sac*I sites, and the resulting plasmid was named as pBSIISK-hFLEX. Then, the sequence of FGL-2A-palmRFP1 was amplified by PCR (primer set P26/P27), and was inserted into pBSIISK-hFLEX through the *EcoR*I/*Sal*I sites, resulting in pBSIISK-FLEX-FGL-2A-palmRFP1. The GFP sequence of pENTR1A-SV40LpA-tTAad-SYN-insulator-TRE-GFP-BGHpA was replaced with the fragment FLEX-FGL-2A-palmRFP1 through the *BamH*I/*Mlu*I sites. The entry vector was finally converted to pAAV2-SynTetOff-FLEX-FGL-2A-palmRFP1 by an LR recombination reaction with pAAV2-DEST(f).

### Production and purification of AAV vectors

pAAVs and two helper plasmids were used for AAV production. pHelper (Stratagene) expresses the adenovirus helper functions (*E2A*, *E4*, and *VA* genes). The other helper plasmid, pBSIISK-R2C1, expressing the replication protein of AAV serotype 2 (Rep2) and the capsid protein of AAV serotype 1 (Cap1), was newly prepared by inserting the following fusion sequences into the *Xho*I/*Not*I sites of pBSIISK: 1) nucleotides 146–2,202 of wild-type AAV2 genome (GenBank accession number, AF043303.1); 2) nucleotides 2,223–4,433 of AAV1 (AF063497.1); and 3) nucleotides 4,438–4,534 of AAV2.

Production and purification of AAV vectors were performed as reported previously [48–50]. Briefly, pAAVs, pHelper, and pBSIISK-R2C1 were cotransfected into HEK293T cells (RCB2202, Riken, Japan) by using polyethylenimine (23966; Polysciences, Inc., Warrington, PA). The medium was replaced 6 h after transfection with Dulbecco’s modified Eagle’s medium (11965–092; Life Technologies) containing 10% fetal bovine serum, 4 mM l-glutamine (25030–081; Life Technologies), 2 mM GlutaMAX^™^ (35050–061; Life Technologies), 0.1 M non-essential amino acids (35050–061; Life Technologies), and 1 mM sodium pyruvate (11360–070; Life Technologies). Cells containing virus particles were collected 72 h after medium replacement. After extraction by three cycles of freeze-and-thaw, the virus particles were purified from a crude lysate of the cells by ultracentrifugation with OptiPrep (AXS-1114542; Axis-Shield, Oslo, Norway) and then concentrated by ultrafiltration with Amicon Ultra-15 Centrifugal Filter Unit with Ultracel-50 membrane (UFC905024; Merck Millipore, Darmstadt, Germany).

We added 4 μL of the virus solution into the HEK293T cells on CELLSTAR^®^ cell culture 12-well plates (665180; Greiner Bio-One, Frickenhausen, Germany) in 1 mL of Dulbecco’s modified Eagle’s medium containing 10% fetal bovine serum, 2 mM l-glutamine, and 0.1 M non-essential amino acids. After two days of incubation, the genomic DNA of each AAV vector was extracted from the infected cells with QIAamp DNA Mini Kit (51304; QIAGEN, Hombrechtikon, Switzerland). Quantitative PCR (qPCR) was performed on a Mini Opticon Real Time PCR System (Bio-Rad, Hercules, CA) with Ssofast^™^ EvaGreen supermix (Bio-Rad) (primer set P28/P29). After 40 amplification cycles with an anneal/extension step at 60°C, the copy number of BGHpA was determined by comparison with the standard curve *via* the Opticon Monitor Software (Bio-Rad), and virus titers (infectious unit/mL, IFU/mL) were adjusted to 1.0 × 10^11^ IFU/mL with Dulbecco's phosphate-buffered saline (14249–95; Nacalai tesque, Kyoto, Japan). The virus solution was stored in aliquots at −80°C until use for delivery to brain tissues.

### Quantification of GFP expression level in Neuro-2a cells

A Neuro-2a cell line (IFO50081; Health Science Research Resources Bank, Osaka, Japan), derived from mouse albino neuroblastoma, was used for the quantification of GFP expression level *in vitro*. Neuro-2a cells were harvested on CELLSTAR^®^ cell culture 12-well plates in 1 mL of minimum essential medium (11095–080; Life Technologies) with 10% fetal bovine serum and 0.1 M non-essential amino acids solution, and 4 μL of virus solution was then added to each well. After 1-week of incubation in three wells with each virus solution, total RNA and DNA were extracted using an AllPrep DNA/RNA Mini Kit (80204; QIAGEN) according to the manufacturer’s protocol. The total RNA and DNA were eluted in 50 and 100 μL of RNase-free water, and 1 and 2 μL were immediately used for analysis, respectively.

Quantitative reverse transcription PCR (qRT-PCR) and qPCR were performed on a Mini Opticon Real Time PCR System with iTaq^™^ Universal SYBR^®^ Green One-Step Kit (172–5150; Bio-Rad) and Ssofast^™^ EvaGreen supermix (172–5200; Bio-Rad), respectively, according to the manufacturer’s instructions (primer set P30/P31). After 40 amplification cycles with an anneal/extension step at 65°C for five samples of each AAV vector, we calculated the copy number of GFP in each reaction by comparison with the standard curve *via* the Opticon Monitor Software. The control reactions without template were included in each assay. The GFP-mRNA/GFP-DNA ratio was used as an index of transcriptional activity.

For comparison of GFP-native fluorescence (NF) intensities by infection with AAV vectors, Neuro-2a cells were cultured on Biocoat^™^ Poly-d-Lysine cover glasses (354087; Corning Life Science, Tewksbury, MA) in the same medium as above, and were incubated for 1 week with 4 μL of virus solutions. The glasses were then removed and fixed with 4% formaldehyde, 0.9% picric acid, and 0.1 M Na_2_HPO_4_ (adjusted to pH 7.0 with NaOH). After washes with phosphate-buffered 0.9% (w/v) saline (PBS; pH 7.4), the glasses were placed upside down on gelatin-coated glass slides with 50% (v/v) glycerol and 2.5% (w/v) triethylenediamine (anti-fading reagent) in PBS. GFP-native fluorescence (GFP-NF) was observed under a TCS SP8 confocal laser scanning microscope with a 25× water-immersion objective lens (HCX PL APO, NA = 0.95; Leica; ~300 cells in total from three glasses). The digital images were saved as 12-bit TIFF files in gray scale without contrast enhancement. The average intensities per pixel of GFP-NF in cells (arbitrary unit; AU) were measured using ImageJ (ver. 1.48; National Institutes of Health; http://imagej.nih.gov/ij).

### Tissue preparation and immunofluorescence staining

The following procedures were performed at room temperature unless stated otherwise. Mice were deeply anesthetized with chloral hydrate (7 mg/10 g body weight) and mounted onto a stereotaxic apparatus. Virus solution (0.2 μL) was injected by pressure through a glass micropipette attached to Picospritzer III (Parker Hannifin Corporation, Cleveland, OH) into the neostriatum of wild mice (1.0 mm anterior to the bregma, 1.8 mm lateral to the midline, and 2.7–2.8 mm deep from the brain surface) or into the S1BF of VIP-Cre mice (1.0 mm posterior to the bregma, 3.0 mm lateral to the midline, and 0.2–0.3 mm deep from the brain surface). The animals were maintained under regular health checks for 1 week and then subjected to transcardial perfusion as described below.

Mice were deeply anesthetized again with chloral hydrate (7 mg/10 g body weight) and transcardially perfused with PBS. The animals were further perfused with 4% (w/v) formaldehyde, 0.1 M Na_2_HPO_4_ (adjusted to pH 7.0 with NaOH), and 0.9% (w/v) picric acid. The brains were then removed and postfixed for 3 h in the same fixative. The brain blocks were cut into 40-μm-thick coronal or sagittal sections on a freezing microtome. Virus-injected brain sections were prepared 1 week after the injection.

Brain sections obtained from wild type mice injected with AAV2/1-SynTetOff-FGL-2A-palmRFP1 were incubated overnight in PBS containing 0.3% Triton^™^ X-100, 0.12% λ-carrageenan, 0.02% sodium azide, and 1% normal donkey serum (PBS-XCD) with 10 μg/mL mouse monoclonal antibody against neuron-specific nuclear protein (NeuN; MAB377; Merck Millipore) or 1 μg/mL rabbit antibody against microtubule-associated protein 2 (MAP2; A0703; Santa Cruz Biotechnology, Santa Cruz, CA). After washes with PBS containing 0.3% Triton^™^ X-100 (PBS-X), the sections were incubated for 2 h with 5.0 μg/mL Alexa Fluor^®^ 568-conjugated goat antibody against mouse IgG (A-11031; Life Technologies) or 5.0 μg/mL Alexa Fluor^®^ 647-conjugated goat antibody against rabbit IgG (A-21245; Life Technologies) in PBS-XCD. Brain sections obtained from VIP-Cre mice injected with AAV2/1-SynTetOff-FLEX-FGL-2A-palmRFP1 were incubated overnight with a mixture of 20 μg/mL anti-GFP chicken antibody (GFP-1020; Aves Labs, Tigard, OR), 1.0 μg/mL anti-mRFP1 rabbit antibody [51], and 1.0 μg/mL anti-VGluT2 guinea pig antibody [52, 53] in PBS-XCD. After washing, the sections were incubated for 2 h with a mixture of 5 μg/mL Alexa Fluor^®^ 488-conjugated goat antibody against chicken IgY (A-11039; Life Technologies), 5 μg/mL Alexa Fluor^®^ 568-conjugated goat antibody against rabbit IgG (A-11039; Life Technologies), and 5 μg/mL Alexa Fluor^®^ 647-conjugated goat antibody against guinea pig IgG (A-21450; Life Technologies) in PBS-XCD. The sections were finally counterstained with 1 μg/mL DAPI (D-1306; Life Technologies) in PBS-X for identification of cortical layers. The sections were mounted onto gelatin-coated glass slides, and coverslipped with 50% (v/v) glycerol and 2.5% (w/v) triethylenediamine (anti-fading reagent) in PBS.

### Image acquisition and measurement of fluorescence intensity

The fluorescence images were acquired under a TCS SP8 confocal laser scanning microscope equipped with a 25× water-immersion objective lens (HCX PL APO, NA = 0.95; Leica) and the pinhole at 1.0 Airy disk unit. GFP and Alexa Fluor^®^ 488, mRFP1 and Alexa Fluor^®^ 568, or Alexa Fluor^®^ 647 was excited with 488, 543, or 633 nm laser beams and observed through 500–580, 590–650, or 660–850 nm emission prism windows, respectively.

The digital images were saved as 12-bit TIFF files in gray scale without contrast enhancement. We measured the average intensities per pixel of GFP-NF in cells (AU) using ImageJ. GFP-NF of ~300 cells from three glasses *in vitro* and that of ~100 neurons in the caudate-putamen (CPu) from three mice were measured for each AAV vector. Cytoarchitecture was determined with reference to NeuN immunoreactivity.

### Statistical analysis

Multiple statistical comparisons were performed by Tukey’s multiple-comparison test after one-way analysis of variance (Prism4.0c; GraphPad Software, San Diego, CA).

## Results

### Gene transduction in Neuro-2a cells with AAV-SynTetOff

**Fig 1:**
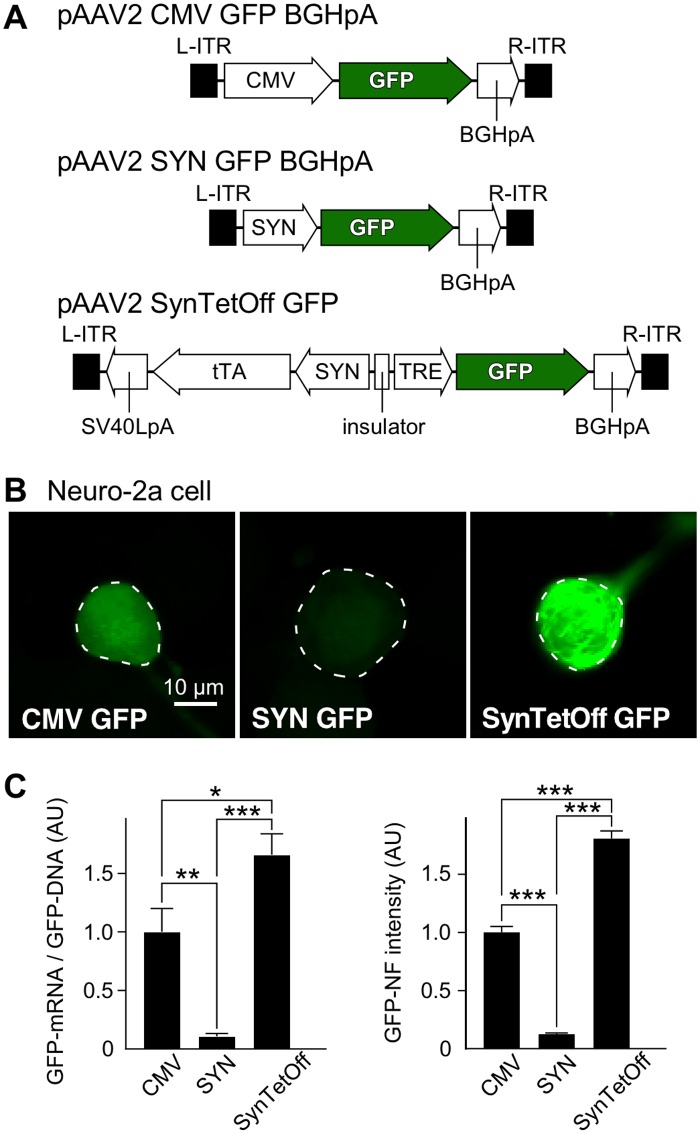
Efficient gene-transduction with AAV-SynTetOff vector *in vitro*. (**A**) Construction of the vector plasmids, pAAV2-CMV-GFP-BGHpA, pAAV2-SYN-GFP-BGHpA, and pAAV2-SynTetOff-GFP. (**B**) One week after infection of Neuro-2a cells with AAV2/1-CMV-GFP-BGHpA, AAV2/1-SYN-GFP-BGHpA, and AAV2/1-SynTetOff-GFP, GFP-NF intensities were measured in the infected cells (outlined by dotted lines with ImageJ). (**C**) The gene-transduction efficiency with AAV2/1-CMV-GFP-BGHpA, AAV2/1-SYN-GFP-BGHpA, or AAV2/1-SynTetOff-GFP was examined quantitatively. The GFP-mRNA/GFP-DNA ratio and the GFP-NF intensity of cells infected with AAV2/1-CMV-GFP-BGHpA were standardized as 1 arbitrary unit (AU). A qRT-PCR assay revealed that GFP-mRNA expression was 1.7- and 15.6-fold higher with AAV2/1-SynTetOff-GFP than with AAV2/1-CMV-GFP-BGHpA and AAV2/1-SYN-GFP-BGHpA, respectively (normalized to GFP-DNA levels). GFP-NF intensities showed similar ratios to AAV2/1-CMV-GFP-BGHpA and AAV2/1-SYN-GFP-BGHpA (factors of 1.8 and 14.4, respectively), indicating that the GFP-NF intensities reflected GFP-mRNA expression levels. Error bars, ± standard error of the mean (SEM). *p < 0.05, **p < 0.01, ***p < 0.001.

In this study, we developed a new AAV vector, “AAV-SynTetOff platform” (Fig 1A); the platform is composed of regulator and response elements separated by the chicken β-globin insulator [44] in a single AAV2 genome. The regulator element expressed tTAad under the control of the SYN promoter [41], whereas the response element produced the reporter protein under the TRE promoter, as reported previously for lentivirus vectors [32]. We also prepared AAV vectors expressing GFP under the CMV promoter and the SYN promoter, i.e., AAV2/1-CMV-GFP-BGHpA and AAV2/1-SYN-GFP-BGHpA (Fig 1A), respectively, as control vectors.

One week after infection of Neuro-2a cells with the AAV vectors, we examined the gene-transduction efficiency by assessing the GFP expression level (Fig 1B). After collecting the infected cells, we performed qRT-PCR for GFP-mRNA and qPCR for GFP-DNA, and normalized the expression level of GFP-mRNA by dividing the amount of GFP-DNA. The GFP-mRNA expression was 1.7- or 15.6-fold higher with AAV2/1-SynTetOff-GFP than with AAV2/1-CMV-GFP-BGHpA or AAV2/1-SYN-GFP-BGHpA, respectively (Fig 1C).

We also randomly selected approximately 300 GFP-expressing cells, and measured the average intensity per pixel of GFP-NF in the cell bodies by using the ImageJ software. With AAV2/1-SynTetOff-GFP, the intensity exhibited a 1.8- or 14.4-fold increase compared with AAV2/1-CMV-GFP-BGHpA or AAV2/1-SYN-GFP-BGHpA, respectively (Fig 1C). The increase of GFP-NF intensity was similar to that observed for GFP-mRNA, suggesting that the fluorescence intensity reflects the mRNA expression level.

### Strong and neuron-specific gene transduction with AAV-SynTetOff in neostriatal neurons

**Fig 2:**
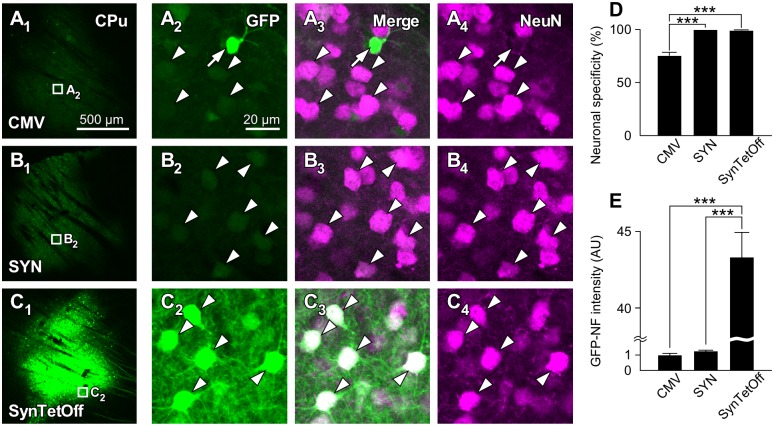
Neuron-specific and high-level transgene expression with the AAV-SynTetOff-GFP vector *in vivo*. (**A**_**1**_**–C**_**4**_) One week after the injection of the AAV vectors, GFP-NF was observed in the caudate-putamen (CPu). Cells infected with the vector AAV2/1-SynTetOff-GFP (C) were more strongly labeled with GFP than those infected with the vectors AAV2/1-CMV-GFP-BGHpA (A) and AAV2/1-SYN-GFP-BGHpA (B). Almost all GFP-positive cells were also immunoreactive for NeuN with AAV2/1-SYN-GFP-BGHpA (B_2_–B_4_, arrowheads) and AAV2/1-SynTetOff-GFP (C_2_–C_4_, arrowheads), whereas some GFP-positive cells were negative for NeuN with the AAV2/1-CMV-GFP-BGHpA vector (A_2_–A_4_, arrow; a putative glial cell). Scale bar in A_1_ applies to A_1_–C_1_; Scale bar in A_2_ applies to A_2_–A_4_, B_2_–B_4_, and C_2_–C_4_. (**D**) Specificities of GFP expression in neostriatal neurons. AAV2/1-SYN-GFP-BGHpA and AAV2/1-SynTetOff-GFP displayed specific expression in neuronal cells, while the expression of GFP with AAV2/1-CMV-GFP-BGHpA was not neuron-specific. (**E**) GFP-NF intensities in neostriatal neurons. The mean GFP-NF intensity with AAV2/1-CMV-GFP-BGHpA was standardized as 1 AU. AAV2/1-SynTetOff-GFP transduced much stronger GFP expression in neurons than AAV2/1-CMV-GFP-BGHpA and AAV2/1-SYN-GFP-BGHpA (factors of 43.3 and 34.3, respectively). Error bars, ± SEM. *p < 0.05, **p < 0.01, ***p < 0.001.

One week after the injection of the AAV vectors into the mouse neostriatum (caudate-putamen, CPu), we observed strong GFP-NF with AAV2/1-SynTetOff-GFP compared with AAV2/1-CMV-GFP-BGHpA and AAV2/1-SYN-GFP-BGHpA (Fig 2A_1_–2C_1_). We randomly selected approximately 300 GFP-expressing cells around the injection sites, and examined the neuronal specificity with NeuN immunoreactivity (Fig 2A_2_–2C_4_). Almost all GFP-expressing cells showed immunoreactivity for NeuN in the CPu with AAV2/1-SynTetOff-GFP and AAV2/1-SYN-GFP-BGHpA (99.4% ± 1.0% and 100% ± 0.0%, respectively, mean ± SD; Fig 2D), indicating that these vectors work specifically in neuronal cells. On the other hand, a significant number of GFP-expressing cells were negative for NeuN immunoreactivity with AAV2/1-CMV-GFP-BGHpA (75.5% ± 5.5%; Fig 2D), since the CMV promoter is a ubiquitous promoter.

We then randomly selected more than 100 GFP-expressing neurons from three mice, and analyzed the expression levels by measuring the average intensity of GFP-NF in the cell bodies. The intensity was 43.3- or 34.3-fold stronger with AAV2/1-SynTetOff-GFP than with AAV2/1-CMV-GFP-BGHpA or AAV2/1-SYN-GFP-BGHpA, respectively (Fig 2E). Although the activity of the CMV promoter was significantly higher than that of the SYN promoter in Neuro-2a cells (Fig 1C) and embryonic mouse spinal cord culture with AAV9 vectors [31], we detected no significant difference between these two promoters *in vivo*. The present findings indicate that the AAV-SynTetOff platform is suitable for strong and neuron-specific gene transduction *in vivo*.

### Clear visualization of neuronal processes with modified GFP

**Fig 3:**
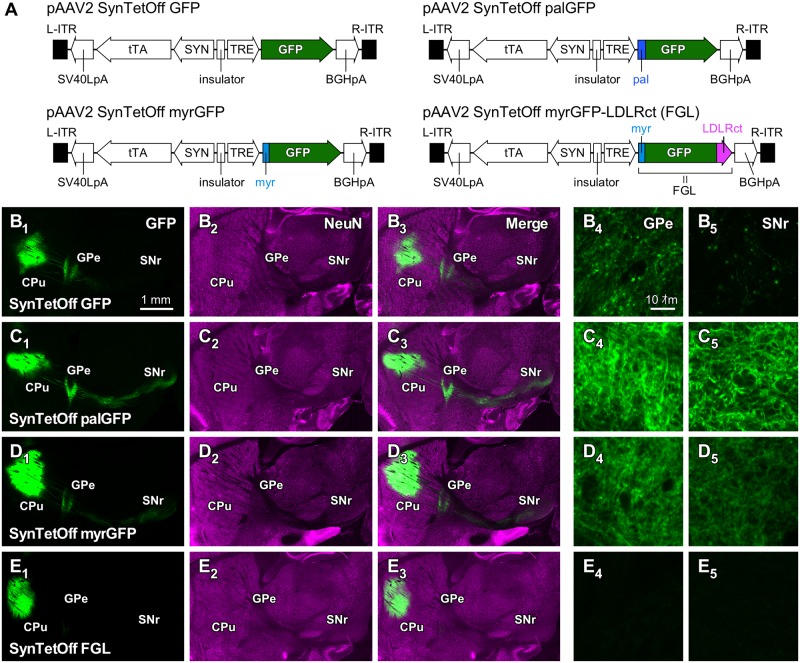
Axon labeling of neostriatal neurons with the AAV-SynTetOff vectors. (**A**) Construction of the vector plasmids, pAAV2-SynTetOff-GFP, pAAV2-SynTetOff-palGFP, pAAV2-SynTetOff-myrGFP, and pAAV2-SynTetOff-FGL. (**B–E**) Sagittal views of brain sections with injections of the AAV vectors. By addition of the palmitoylation site of the GAP-43 N-terminus (palGFP; C) or the myristoylation/palmitoylation site of the Fyn N-terminus (myrGFP; D), axon fibers in the GPe and SNr were more clearly visualized than with GFP without membrane-targeting signal (B). On the other hand, when a somatodendritic-targeting signal, LDLRct, was added to the C-terminus of myrGFP (FGL; E), axon fiber labeling in the GPe and SNr was mostly suppressed (E_4_, E_5_). Scale bar in B_1_ applies to B_1_–E_1_, B_2_–E_2_, and B_3_–E_3_. Scale bar in B_4_ applies to B_4_–E_4_ and B_5_–E_5_.

To visualize axon fibers efficiently, we modified GFP by adding the following plasma membrane-targeting signals: a palmitoylation signal (palGFP) [32, 35–39] or a myristoylation/palmitoylation signal (myrGFP) [39] (Fig 3A). One week after the injection of AAV2/1-SynTetOff-GFP, AAV2/1-SynTetOff-palGFP, and AAV2/1-SynTetOff-myrGFP into the mouse CPu, we observed GFP-NF around the injection sites as well as the projection targets such as the external segment of the globus pallidus (GPe) and the substantia nigra pars reticulata (SNr; Fig 3B_1_–3D_3_). Strong GFP-NF was observed around the injection sites with these AAV vectors. GFP-NF with AAV2/1-SynTetOff-GFP was weak in the GPe and very weak in the SNr (Fig 3B_4_ and 3B_5_). On the other hand, the axon fibers of neostriatal neurons, which are presumably medium-sized spiny neurons, were clearly visualized with membrane-targeted GFP, especially with palGFP (Fig 3C_4_–3D_5_). These results indicate that the membrane-targeted GFP can be effectively used for visualizing axonal processes.

We then attached the somatodendrite-targeting signal, LDLRct, to the C-terminus of myrGFP (FGL; Fig 3A) [39, 40]. Although intense GFP-NF was observed around the injection sites, almost no signal was found in the GPe and SNr (Fig 3E_1_–3E_5_). As reported in a previous study with a lentivirus vector [39], the somatodendritic membrane-targeted GFP efficiently labeled the somatodendritic domains of the infected neurons, but not the axonal structures, with AAV-SynTetOff-FGL.

**Fig 4:**
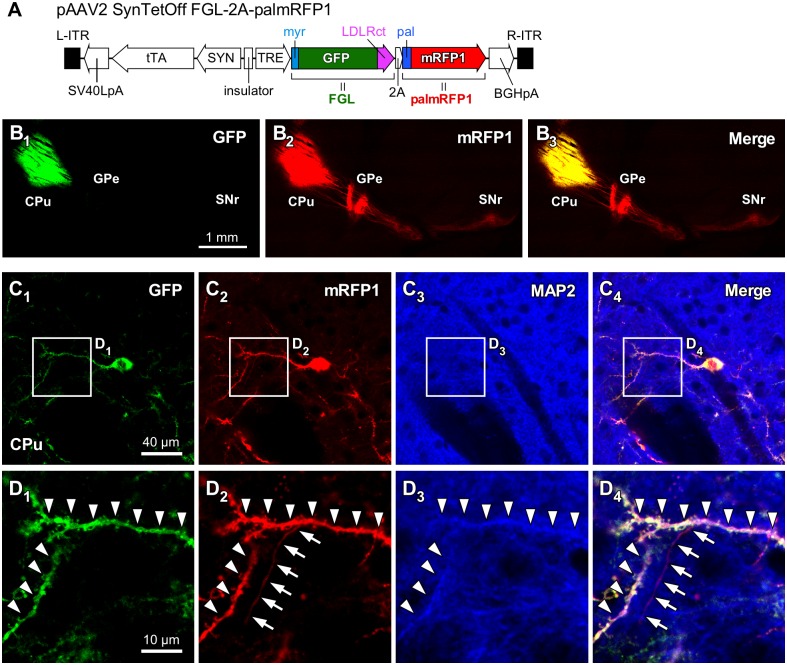
Dual-color labeling of the somatodendritic and axonal structures of neostriatal neurons with the AAV-SynTetOff vectors. (**A**) Construction of the vector plasmid, pAAV2-SynTetOff-FGL-2A-palmRFP1. (**B**) Sagittal view of brain sections with injection of AAV2/1-SynTetOff-FGL-2A-palmRFP1 into the CPu. This AAV vector expressed both FGL and palmRFP1 in the infected neurons. GFP-NF was restricted to the CPu, and mRFP1-NF was observed not only in the CPu but also the GPe and SNr, where neostriatal neurons project. (**C**) After immunofluorescence staining for MAP2 (blue), the sections were observed under a confocal laser scanning microscope in the CPu. (**D**) High-magnification images in (C). Arrowheads and arrows indicate the dendritic and axonal structures, respectively. Scale bar in B_1_ applies to B_1_–B_3_. Scale bar in C_1_ applies to C_2_–C_4_. Scale bar in D_1_ applies to D_2_–E_4_.

To demonstrate the effects of the membrane- and/or dendrite-targeting signals, we generated an AAV vector that expressed both FGL and palmRFP1 (Fig 4A). One week after injection of this vector, we observed the distribution of GFP-NF and mRFP1-NF. As expected, GFP-NF was restricted within the CPu, whereas mRFP1-NF was clearly observed not only in the CPu but also in the GPe and SNr (Fig 4B_1_–4B_3_). We further performed immunofluorescence staining for MAP2, which is considered a marker for dendrites (Fig 4C_1_–4D_4_). Dendrites labeled with both GFP and mRFP1 displayed immunoreactivity for MAP2 (Arrowheads in Fig 4D). On the other hand, the axon fiber was visualized with mRFP1 but not GFP, and was negative for MAP2 immunoreactivity (Arrows in Fig 4D). These results clearly show that the somatodendritic-targeting signal effectively localizes GFP and that the axons can be distinguished by the presence of mRFP1 signal and the absence of GFP signal.

This vector, AAV2/1-SynTetOff-FGL-2A-palmRFP1, might be useful for elucidating the differential distributions of somatodendritic and axonal structures without reconstruction of the infected neurons. In the subsequent experiment, we applied this vector to one of the neocortical GABAergic neurons, to demonstrate the usefulness of the dual-color labeling.

### Differential distributions of dendrites and axons of VIP+ neurons

VIP+ neurons are one of the subclasses of GABAergic neurons in the neocortex, and are mainly distributed in layer (L) L2/3 [54–56]. Most VIP+ neurons in the neocortex bidirectionally extend their dendrites in the vertical orientation, and the axon fibers run vertically and translaminarly across cortical layers. On the basis of the characteristic features of the dendrites and axons, VIP+ neurons in the neocortex have been classified as bipolar/modified bipolar/bitufted cells and double bouquet cells [57–60].

Contrary to the vertical arborizations, the somatodendritic and axonal distributions of VIP+ neurons in the tangential/horizontal direction are yet to be clarified. In a previous immunohistochemical study, VIP immunoreactivity was unevenly observed in L4 of the rodent S1BF [61, 62]. There are “barrels” and inter-barrel “septa” in L4 of the S1BF, and each barrel-related column in the rodent S1BF receives input from the corresponding vibrissa of the contralateral whisker pad [63–67]. While the immunoreactivity for vesicular glutamate transporter 2 (VGluT2), which is a marker for thalamocortical axon terminals [68–70], is higher in barrels than in septa [71–73], the immunoreactivity for VIP is higher in septa than in barrels [61, 62]. By applying the dual-color labeling method described above, we analyzed the somatodendritic and axonal distributions of L2/3 VIP+ neurons in the S1BF to identify whether the higher immunoreactivity for VIP in septa is derived from dendrites or axons.

**Fig 5:**
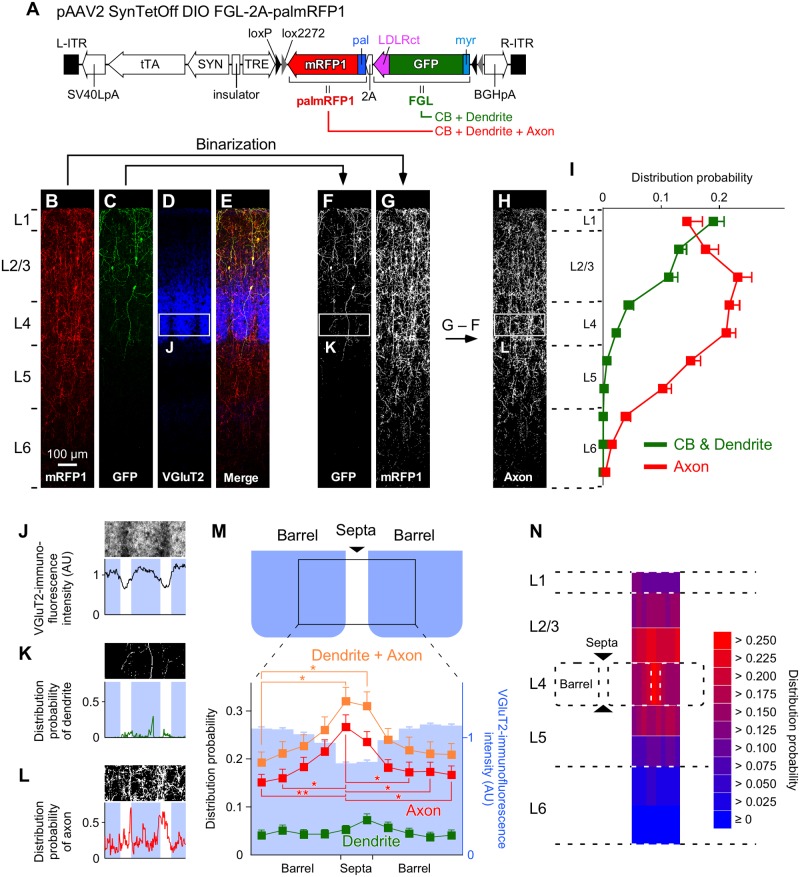
Differential distributions of the dendrites and axons of L2/3 VIP+ neurons in the S1BF. (**A**) Construction of the vector plasmid, pAAV2-SynTetOff-FLEX-FGL-2A-palmRFP1. (**B–E**) Coronal sections immunostained for mRFP1 (B), GFP (C), and VGluT2 (D). Scale bar in B applies to B–E. (**F**, **G**) Binarization of the images for GFP and mRFP1. The binary image of GFP signals (F) represents the somatodendritic distribution of L2/3 VIP+ neurons. (**H**) Axonal distribution was estimated by subtracting the binarized image for GFP (F) from that for mRFP1 (G). (**I**) The distribution probabilities of the somatodendrites and axons of L2/3 VIP+ neurons across cortical layers. (**J**) VGluT2-immunofluorescence intensity in the tangential direction in L4 of the S1BF. Blue indicates barrel locations. (**K**, **L**) The distribution probabilities of the dendrites (K) or axons (L) of L2/3 VIP+ neurons in L4 of the S1BF. (**M**) Mean distribution probabilities of the dendrites and/or axons of L2/3 VIP+ neurons in L4 of the S1BF. The probabilities are plotted between the centers of adjacent barrels. The tangential width was divided into 10 bins. Blue bars represent VGluT2 immunofluorescence intensities. (**N**) Axonal distributions of L2/3 VIP+ neurons in the S1BF. The vertical widths of L2/3, L5, and L6 were divided by a factor of 2 at the center of each layer. The distribution probability of the axons in L4 was higher in septa than in barrels, whereas those in the other layers were not significantly different between the barrel- and septa-related columns. Error bars, ± SEM. *p < 0.05, **p < 0.01.

One week after the injection of AAV2/1-SynTetOff-FLEX-FGL-2A-palmRFP1 into the superficial layer in the S1BF of VIP-Cre knock-in mice, we observed the immunoreactivities for GFP and mRFP1 under a confocal laser scanning microscope, and selected three samples in which the infection was restricted to L2/3 VIP+ neurons (Fig 5A–5E). The Somata and dendrites of L2/3 VIP+ neurons were labeled with GFP, whereas their axons as well as somatodendrites were visualized with mRFP1 (Fig 5B and 5C). After binarization of the images of immunofluorescence-stained sections for GFP and mRFP1 (Fig 5F and 5G), the binary image of GFP was subtracted from that of mRFP1 (Fig 5H). The binarized signals of GFP represented the somatodendritic distribution of L2/3 VIP+ neurons (Fig 5F), whereas the subtracted image represented their axonal distribution (Fig 5H).

The somatodendritic and axonal distributions were first quantified vertically from the pia mater to the white matter (Fig 5I). The distribution of the dendrites of L2/3 VIP+ neurons was largely restricted to the regions between L1 and L4, whereas the axons were present from L1 to L6, consistent with previous studies [57–59]. Given the vertical arborization of L2/3 VIP+ neuron axon fibers within a small tangential expansion [74], their outputs diverge to the deep layer within the vertical modules (e.g. a columnar structure), though they receive synaptic inputs in the upper layer.

We subsequently examined the dendritic and axonal distributions of L2/3 VIP+ neurons in the tangential direction (Fig 5J–5N). In L4, the distribution of dendrites was not different between barrels and septa, whereas axons obviously preferred septa to barrels (Fig 4M). On the other hand, axonal distribution in other layers was not different between barrel- and septa-related columns (Fig 5N). These results indicate that L2/3 VIP+ neurons send axons to L1–6, with preferential placement in L4 through septa.

## Discussion

### High-level gene transduction with the AAV-SynTetOff vector

AAV vectors are one of the most useful gene-transfer systems, and a variety of AAV vectors have been developed with ubiquitous or neuron-specific promoters, including the CMV or SYN promoters, to improve the expression levels and/or cell-type specificities [75–78]. In the present study, we incorporated a neuron-specific promoter (SYN promoter) and the Tet-Off system into a single vector, and successfully achieved both neuronal specificity and strong gene transduction. The SYN promoter expressed tTAad specifically in neuronal cells, and then, the TRE promoter induced strong transduction of the reporter protein. The Tet-Off system is known to amplify transcriptional activities [32, 79, 80]. The AAV-SynTetOff vector might be suitable for the efficient labeling of neuronal cells, since the vector can produce large amounts of protein in the infected cells. In addition, replacing the SYN promoter sequence with the desired sequence will enable the induction of high-level expression of transgene(s) in other type(s) of cells.

We assessed the gene-transduction efficiency of the AAV-SynTetOff vector by calculating the ratio of GFP-mRNA/GFP-DNA and GFP-NF intensity in Neuro-2a cells, and demonstrated that the ratio was comparable to that of GFP-NF intensity; the ratio with the AAV-SynTetOff vector was 1.7- and 15.6-fold higher than that with the CMV and SYN promoters, respectively, and GFP-NF intensity with the AAV-SynTetOff vector was 1.8- and 14.4-times higher than that with the CMV and SYN promoters, respectively. Since the GFP-mRNA/GFP-DNA ratio and GFP-NF intensity are assumed to reflect the transcriptional activity and protein expression level, the gene-transduction efficiency can be estimated by measuring GFP-NF intensity in the infected cells. However, the *in vitro* and *in vivo* gene-transduction efficiencies estimated by using GFP-NF were different. While the GFP-NF intensity in the neostriatal neurons infected with AAV-SynTetOff was 43.3-fold higher than that observed with the conventional AAV vector containing the CMV promoter, the difference in GFP-NF intensity in Neuro-2a cells between the two vectors was only 1.8-fold. This discrepancy might be attributable to the differences in cell types between neostriatal neurons and Neuro-2a cells. The gene-transduction efficiency of the AAV-SynTetOff vector may be different in various cell types, and this needs to be confirmed in every experiment.

In our previous studies, we developed lentivirus vectors equipped with the Tet-Off system [32, 33], and achieved high-level gene transduction compared with conventional lentivirus vectors. However, it was technically difficult to prepare lentivirus vectors that carried both tTAad and the TRE promoter within a single backbone in high titer [32, 33]. The preparation of AAV vectors in high titer is easier than preparing lentivirus vectors [81]. In addition, AAV vectors are more efficient in gene transfer to central neurons than lentivirus vectors pseudotyped with the VSV glycoprotein (VSV-G) [81]. Therefore, the AAV-SynTetOff vectors may be more promising than the single Tet-Off lentivirus vectors for transgene delivery to central neurons.

### Dual fluorescence labeling with single AAV-SynTetOff vectors

Neostriatal projection neurons, which account for 90%–95% of all neostriatal neurons, comprise both direct and indirect pathway neurons [82]; direct pathway neurons directly project to the output nuclei, the internal segment of the globus pallidus (GPi) and SNr, though some of them send axon collaterals to the GPe [83], while the indirect pathway neurons send axons exclusively to the GPe. In the present study, we injected virus vectors into the CPu to evaluate the efficiency of axonal labeling in the projection neurons.

The AAV-SynTetOff vectors expressing membrane-targeting signal-attached GFP (palGFP or myrGFP) visualized the axon fibers of neostriatal projection neurons more efficiently than the vector without the membrane-targeting signal (Fig 3B–3D). On the other hand, as reported previously [39], the dendrite-targeting signal, LDLRct, strongly suppressed the axonal labeling of neostriatal projection neurons infected by AAV2/1-SynTetOff-FGL (Fig 3E). In the present study, we generated the vector AAV2/1-SynTetOff-FGL-2A-palmRFP1, which expresses both FGL and palmRFP1, to visualize the somatodendritic and axonal structures in different colors (Fig 3F). After injection of the vector into the CPu, the axons of neostriatal projection neurons were labeled only by palmRFP1, whereas the somatodendritic structures were visualized by FGL and palmRFP1 (Fig 3G). Thus, this vector might be useful to distinguish the somatodendritic and axonal arborizations of the infected neurons by observing fluorescent signals.

We then applied this dual fluorescence labeling method to VIP+ neurons in the neocortex, which account for only a small fraction of neocortical GABAergic neurons [54, 55, 84–87], to analyze the somatodendritic and axonal distribution of L2/3 VIP+ neurons at the population level. After injection of AAV2/1-SynTetOff-FLEX-FGL-2A-palmRFP1 into the S1BF of VIP-Cre knock-in mice, the somatodendritic structures of L2/3 VIP+ neurons were labeled with FGL, whereas the whole structure, including the axons, were visualized by palmRFP1 (Fig 5B and 5C). After binarization of the images, the somatodendritic and axonal distributions were quantitatively analyzed through the cortical depth (Fig 5F–5I). To date, to quantitatively analyze the somatodendritic and axonal distributions, it is necessary to trace and reconstruct the dense dendritic and axonal ramifications of each neuron, and the reconstruction of many single neurons is laborious and impractical. The present method, which involves dual fluorescence labeling by the AAV vector, would be valuable to clarify the information flow from dendrites to axons at the population level without tracing and reconstruction of a particular subset of neurons.

### Somatodendritic and axonal arborizations of L2/3 VIP+ neurons in the S1BF

VIP+ neurons are now attracting a great deal of attention in the study of the neocortical circuit. VIP+ neurons in the neocortex potentiate pyramidal cell excitability by inhibiting other types of inhibitory neurons [88–92]. Through this “disinhibitory circuit,” VIP+ neurons are assumed to translaminarly trigger cortical activation within a columnar or subcolumnar structure to facilitate local cortical processing and sensory responses.

The immunoreactivity for VIP is higher in septa than in barrels of the S1BF [61, 62]. Since immunostaining for VIP labels the somata, dendrites and axons of VIP+ neurons, it has not yet been determined which structures, the somatodendrites or axons of VIP+ neurons, prefer septa to barrels. To address this question, we injected the vector AAV2/1-SynTetOff-FLEX-FGL-2A-palmRFP1 into the S1BF of VIP-Cre knock-in mice, and analyzed the somatodendritic and axonal domains of L2/3 VIP+ neurons.

Vertical dendritic expansion was largely restricted to the regions between L1 and L4, whereas axons were present from L1 to L6 (Fig 5I), consistent with a previous study [59]. We then identified septa-preferential axonal arborization of L2/3 VIP+ neurons. Their dendritic arborization showed no significant difference between barrels and septa, indicating that L2/3 VIP+ neurons receive inputs from L4 without distinction between barrels and septa. On the other hand, L2/3 VIP+ neurons sent axon fibers significantly more densely to septa than to barrels.

The thalamocortical projection from the anterior subdivision of the posteromedial nucleus and the corticocortical projections originating from other cortical areas such as the primary motor cortex specifically target septa [93–95]. These paralemniscal projections and interareal connections are considered crucial for the context-dependent sensory processing of pyramidal cells evoked by tactile stimulation and sensorimotor integration [96–98]. Therefore, the septa-preferential output of L2/3 VIP+ neurons might affect these integrative inputs in septa of the S1BF.

### Future direction

In the present study, we demonstrated high-level expression of transgenes with the AAV-SynTetOff vectors and efficient labeling of central neurons. Furthermore, by attaching the membrane-targeting and/or dendrite-targeting signals, the somatodendritic and/or axonal structures of the infected neurons were clearly visualized. Recently, many kinds of tissue-clearing methods have been developed, such as Scale [99, 100], CUBIC [101, 102], CLARITY [103, 104], SeeDB [105, 106], DISCO [107–109], etc. Although these techniques enable us to perform three-dimensional imaging with whole brain or thick-slice samples in large-scale, the intensity of fluorescent signal is critical for deep brain imaging and comprehensive analysis of neuronal structures [99]. The AAV-SynTetOff vectors, which achieve high-level expression of reporter protein(s), would prompt the morphological analysis of neuronal circuits and large-scale connectomic mapping with these tissue-clearing methods.

## Supporting Information

S1 Table Primers and oligonucleotides used in the present study. (DOCX)
