# Supplementary material for: A Single Vector Platform for High-Level Gene Transduction of Central Neurons: Adeno-Associated Virus Vector Equipped with the Tet-Off System
Source: PLoS One. 2017 Jan 6;12(1):e0169611. doi: 10.1371/journal.pone.0169611 (PMC5217859; doi:10.1371/journal.pone.0169611)
Supplement: S1 Table — (DOCX) [file pone.0169611.s001.docx]

**Supporting Information**

**A Single Vector Platform for High-Level Gene Transduction of Central Neurons: Adeno-Associated Virus Vector Equipped with the Tet-Off System**

Jaerin Sohn, Megumu Takahashi, Shinichiro Okamoto, Yoko Ishida, Takahiro Furuta, and Hiroyuki Hioki

**S1 Table. Primers and oligonucleotides used in the present study.**

| P1 | 5′-TAGTTATTAATAGTAATCAA-3′ |
| --- | --- |
| P2 | 5′-GATCTGACGGTTCACTAAAC-3′ |
| P3 | 5′-CTGCAGAGGGCCCTGCGTAT-3′ |
| P4 | 5′-CGCCGCAGCGCAGATGGTCG-3′ |
| P5 | 5′-TCGA**GCCACC**ATGGTGAGCAAGGGCGAGGA-3′ |
| P6 | 5′-TTACTTGTACAGCTCGTCCA-3′ |
| P7 | 5′-AAAAGAATTCCTGTGCCTTCTAGTTGCCAG-3′ |
| P8 | 5′-TTTTGGATCCCCATAGAGCCCACCGCATCC-3′ |
| P9 | 5′-AAAAACGCGTACAAGTTTGTACAAAAAAGC-3′ |
| P10 | 5′-TTTTCACGTGACCACTTTGTACAAGAAAGC-3′ |
| P11 | 5′-AAAAACGCGTACCACTTTGTACAAGAAAGC-3′ |
| P12 | 5′-TTTTCACGTGACAAGTTTGTACAAAAAAGC-3′ |
| P13 | 5′-AAAAAGATCTCAGACATGATAAGATACATT-3′ |
| P14 | 5′-TTGCGGCCGCTACCACATTTGTAGAGGTTT-3′ |
| P15 | 5′-GGATCCGTCGACCGCGCCGCCCCGGGCGGCTCGCTGCTGCCCCCTAGCGGGGGAGGGACGTAATTACATCCCTGGGGGCTTTGGGGGGGCTCGAGGCGGCCGCGTCGAC-3′ |
| P16 | 5′-GTCGACGCGGCCGCCTCGAGCCCCCCCAAAGCCCCCAGGGATGTAATTACGTCCCTCCCCCGCTAGGGGGCAGCAGCGAGCCGCCCGGGGCGGCGCGGTCGACGGATCC-3′ |
| P17 | 5′-AAAACTCGAGCTGCAGAGGGCCCTGCGTAT-3′ |
| P18 | 5′-TTTTAGATCTTACCACATTTGTAGAGGTTT-3′ |
| P19 | 5′-AAAAGGATCC**GCCACC**ATGCTGTGCTGTATGCGAAG-3′ |
| P20 | 5′-TTTTACGCGTTTACTTGTACAGCTCGTCCA-3′ |
| P21 | 5′-AAAAGGATCC**GCCACC**ATGGGCTGTGTGCAATGTAA-3′ |
| P22 | 5′-TTTTACGCGTTCATGCCACATCGTCCTCCA-3′ |
| P23 | 5′-GGGCCCTGGGTTGGACTCCACGTCTCCCGCCAACTTGAGAAGGTCAAAATTCAAAGTCTGTTTCACTGCCACATCGTCCTCCAGGC-3′ |
| P24 | 5′-GTGAAACAGACTTTGAATTTTGACCTTCTCAAGTTGGCGGGAGACGTGGAGTCCAACCCAGGGCCCATGCTGTGCTGTATGAGAAG-3′ |
| P25 | 5′-TTTTACGCGTTTAGGCGCCGGTGGAGTGGC-3′ |
| P26 | 5′-AAAAGTCGAC**GCCACC**ATGGGCTGTGTGCAATGTAA-3′ |
| P27 | 5′-TTTTGAATTCTTAGGCGCCGGTGGAGTGGC-3′ |
| P28 | 5′-GACAGCAAGGGGGAGGATTG-3′ |
| P29 | 5′-GCCAACTCCATCACTAGGGGTTC-3′ |
| P30 | 5′-AAGCTGACCCTGAAGTTCATCTGC-3′ |
| P31 | 5′-CTTGTAGTTGCCGTCGTCCTTGAA-3′ |
| FLEX | 5′-GGTACCTTCGAAGGATCCCCATAACTTCGTATAAAGTATCCTATACGAAGTTATATCAAAATAGGAAGACCAAGTCTTCACCATCGACCCGAATTGCCAAGCATCACCATCGACCCATAACTTCGTATAGCATACATTATACGAAGTTATCTGTGAGTGAATTCGTCGACGATATCCTGCAGATAACTTCGTATAGGATACTTTATACGAAGTTATCATTGGGATTCTTCCTATTTTGATCCAAGCATCACCATCGACCCTCTAGTCCAGATCTCACCATCGACCCATAACTTCGTATAATGTATGCTATACGAAGTTATGTCCCTCGAAGAGGTTCACGCGTGCGGCCGCGAGCTC-3′ |

Bold characters indicate the Kozak consensus sequence. Underlined sequences indicate the restriction sites for *BamH*I in P8, P15, P16 and P19 and P21, *Bgl*II in P13 and P18, *EcoR*I in P7 and P27, *Mlu*I in P9, P11, P20, P22 and P25, *Not*I in P14, *PmaC*I in P10 and P12, *Sal*I in P15, P16 and P26, and *Xho*I in P17. The sequence of FLEX contains multiple unique restriction sites.
